# Balancing Health and Sustainability: Assessing the Benefits of Plant-Based Diets and the Risk of Pesticide Residues

**DOI:** 10.3390/nu17040727

**Published:** 2025-02-19

**Authors:** Alexandra Andreea Botnaru, Ancuta Lupu, Paula Cristina Morariu, Oana Lelia Pop, Alin Horatiu Nedelcu, Branco Adrian Morariu, Oana Cioancă, Maria Luisa Di Gioia, Vasile Valeriu Lupu, Liliana Avasilcai, Oana Maria Dragostin, Madalina Vieriu, Ionela Daniela Morariu

**Affiliations:** 1 Faculty of Pharmacy, “Grigore T. Popa” University of Medicine and Pharmacy, 700115 Iasi, Romania; botnaru.alexandra@yahoo.com (A.A.B.); oana.cioanca@umfiasi.ro (O.C.); liliana.avasilcai@umfiasi.ro (L.A.); madalina.vieriu@umfiasi.ro (M.V.); ionela.morariu@umfiasi.ro (I.D.M.); 2 Department of Environmental and Food Chemistry, “Grigore T. Popa” University of Medicine and Pharmacy, 700115 Iasi, Romania; 3 Faculty of General Medicine, “Grigore T. Popa” University of Medicine and Pharmacy, 700115 Iasi, Romania; anca_ign@yahoo.com (A.L.); alin_nedelcu@yahoo.com (A.H.N.); morariubranco@gmail.com (B.A.M.); valeriulupu@yahoo.com (V.V.L.); 4 Department of Pediatrics, “Grigore T. Popa” University of Medicine and Pharmacy, 700115 Iasi, Romania; 5 Department of Internal Medicine, “Grigore T. Popa” University of Medicine and Pharmacy, 700115 Iasi, Romania; 6 Department of Food Science, University of Agricultural Sciences and Veterinary Medicine, 400372 Cluj-Napoca, Romania; oana.pop@usamvcluj.ro; 7 Department of Morpho-Functional Science I, “Grigore T. Popa” University of Medicine and Pharmacy, 700115 Iasi, Romania; 8 Department of Pharmacognosy, “Grigore T. Popa” University of Medicine and Pharmacy, 700115 Iasi, Romania; 9 Dipartimento di Farmacia, Salute e Scienze della Nutrizione, Università della Calabria, Arcavacata di Rende, 87036 Cosenza, Italy; ml.digioia@unical.it; 10 Research Centre in the Medical-Pharmaceutical Field, Faculty of Medicine and Pharmacy, “Dunarea de Jos” University of Galati, 800010 Galati, Romania; oana.dragostin@ugal.ro; 11 Department of Analytical Chemistry, “Grigore T. Popa” University of Medicine and Pharmacy, 700115 Iasi, Romania

**Keywords:** vegetarian diet, food safety, sustainable diet, pesticide residue, pesticide toxicity, pesticide removal, human health

## Abstract

The increased consumption of fruit and vegetables is essential for moving towards a healthier and more sustainable diet. Vegetarian diets are gaining in popularity due to their environmental and health implications; however, there is a need for additional research investigating pesticide residues in these foods. It is increasingly recognized that the global food system must prioritize nutritional quality, health, and environmental impact over quantity. Food contaminants, including pesticides, mycotoxins, and heavy metals, pose a substantial threat to food safety due to their persistent nature and harmful effects. We conducted a literature search utilizing four distinct databases (PubMed, Google Scholar, NIH, ScienceDirect) and several combinations of keywords (pesticides, food, vegetarian diet, toxicity, sustainable, removal). Consequently, we selected recent and relevant studies for the proposed topic. We have incorporated articles that discuss pesticide residues in food items, particularly in plant-based products. This study rigorously analyzes the harmful environmental impacts of pesticides and ultimately provides sustainable solutions for their elimination or reduction, along with environmentally sound alternatives to pesticide use. This study concludes that the transition towards sustainable agriculture and food production is essential for reducing pesticide residues in food, thereby protecting human health, wildlife populations, and the environment. This paper argues for the urgent need to transform global food systems to prioritize health and sustainability.

## 1. Introduction

An optimal diet should enhance health, which is commonly defined as a state of complete physical, psychological, and social well-being, rather than being only an absence of disease, though it is widely recognized that our dietary selections profoundly affect both our health and the environment. In response to the rising incidence of chronic illnesses, the development of new approaches for their prevention and treatment is growing, one of which requires compliance with specific eating habits [1]. Plant-based diets have gained heightened interest due to multiple studies demonstrating the health and environmental sustainability benefits that are linked to dietary patterns characterized by reduced meat consumption and an elevated intake of fruits and vegetables, legumes, whole grains, nuts, and seeds [2].

Vegetables are abundant in minerals, vitamins, antioxidants, and dietary fiber, but are low in fat, soluble sugars, and calories, making them an essential element of a nourishing diet. The consumption of vegetables has been extensively demonstrated to diminish the risk of many malignancies, heart diseases, and total mortality. Moreover, a diet consisting primarily of plant-derived foods, which includes vegetables, is ecologically sustainable and resource-efficient during its production process, therefore promoting the United Nations Sustainable Development Goals [3].

Despite vegetables playing a crucial nutritional role in health, extended exposure to pesticide-contaminated produce may result in detrimental health consequences [4]. An excessive intake of chemically polluted fruits and vegetables, particularly those that may bioaccumulate in human organs, may result in chronic diseases [5]. Furthermore, some food products might contain the residues of drugs, hence presenting health hazards [6,7]. Therefore, determining contamination in food products to ensure food safety is a real challenge [8]. The unregulated application of pesticides on fruits and vegetables for the prevention of insect damage may lead to residual concentrations beyond the maximum residue limits (MRLs), therefore affecting human health [9]. Authorities establish MRLs for pesticides in food to help reduce these hazards. Nonetheless, the implementation of these standards and routine food testing are both essential for consumer protection.

In recent years, the application of pesticides has significantly escalated, primarily to combat detrimental organisms that impact vital crops. The application of these pesticides must be properly quantified and regulated to avoid detrimental impacts on humans, non-target fauna, and the environment. An optimal pesticide must be target-specific, affecting just the pest species of concern, without causing unintended consequences for other exposed organisms. Achieving absolute selectivity to only affect the target organism is difficult, as most pesticides provide risks to both humans and non-target species. The inadequate use of pesticides can result in the contamination of several environmental components, including soil, water supplies, air, vegetation, and wildlife. The study of food contaminant toxicity is crucial for maintaining food safety, evaluating possible dangers, and developing suitable food safety regulations [10,11].

## 2. Vegetarian Diet

Vegetarian diets are classified into five primary categories based on their dietary exclusions or inclusions: vegans (who consume exclusively plant-based foods), lacto-vegetarians (who follow a plant-based diet that excludes dairy), ovo-vegetarians (who maintain a plant-based diet but consume eggs), lacto-ovo-vegetarians (incorporate both dairy products and eggs alongside plant-based foods), and pesco-vegetarians (omit meat and poultry, dairy, and eggs, but include fish). Research indicates that a vegan diet had the highest nutritional quality score according to the Healthy Eating Index 2010. Processed foods have consistently been seen as harmful, whereas the intake of raw foods has been encouraged [12,13].

Vegan diets are significantly richer in dietary fiber and vitamins (C, E, B9), while minerals such as magnesium and potassium are also more abundant. These diets are also fortified with antioxidants, including flavonoids and carotenoids, providing anti-inflammatory and anticancer benefits, along with beta-carotene. Moreover, plant-based diets are linked to a lower incidence of chronic conditions like cardiovascular disease, cancer, diabetes, obesity, and osteoporosis [14]. The EAT-Lancet Commission recently recommended a daily intake of 300 g per capita of vegetables to promote health [3].

High-income nations, including the United States of America, Israel, Australia, and the United Kingdom, have seen a rise in the acceptance of plant-based diets in recent years. For instance, during a ten-year period, the percentage of individuals in the United Kingdom who consume plant-based substitutes to meat and dairy almost doubled, from 6.7% to 13.1% [15]. In Europe, the estimated percentage of vegans ranges from 1% to 10% [16]. Recent studies revealed that the majority of people in the United States are familiar with plant-based diets, and several individuals expressed a desire to acquire further knowledge about them. Although a limited number considered themselves vegetarians or vegans, characterized by the omission of all or certain animal products, over 20% indicated an increased consumption of plant-based meals. Consequently, a trend towards increased plant-based consumption may be observed among adults in the United States, regardless of the low adoption rates of vegetarian and vegan diets [1]. Conversely, the younger generation often eats out or ignores breakfast and fails to consume adequate fruits and vegetables [3].

Sustainable healthy diets, as defined by the World Health Organization (WHO) and the Food and Agriculture Organization (FAO), are complex, addressing several sustainability dimensions, including human health, environmental effects, and cultural acceptance [17]. Each nation has its own definitions and standards for sustainable and healthful diets. To help nations create food policies that encourage a shift toward nutrient-dense, environmentally sustainable, and culturally acceptable diets, the FAO and the WHO published a set of guidelines. To enhance nutritional health, dietary patterns prioritizing a varied array of unprocessed and minimally processed plant foods are recommended, accompanied by the moderate consumption of animal products and the restricted intake of highly processed foods. Sustainable eating standards aim to mitigate environmental impacts by regulating greenhouse gas emissions, water consumption, and chemical pollution, safeguarding biodiversity, and minimizing plastic food packaging [18].

This dietary change would moderately reduce fertilizer use and would also diminish, albeit to a lower degree, the usage of crops and freshwater resources. Adopting a balanced vegetarian diet, particularly in industrialized nations, may serve as an effective approach to mitigating environmental deterioration within the food system and conserving the planet’s resources [19].

**Figure 1:**
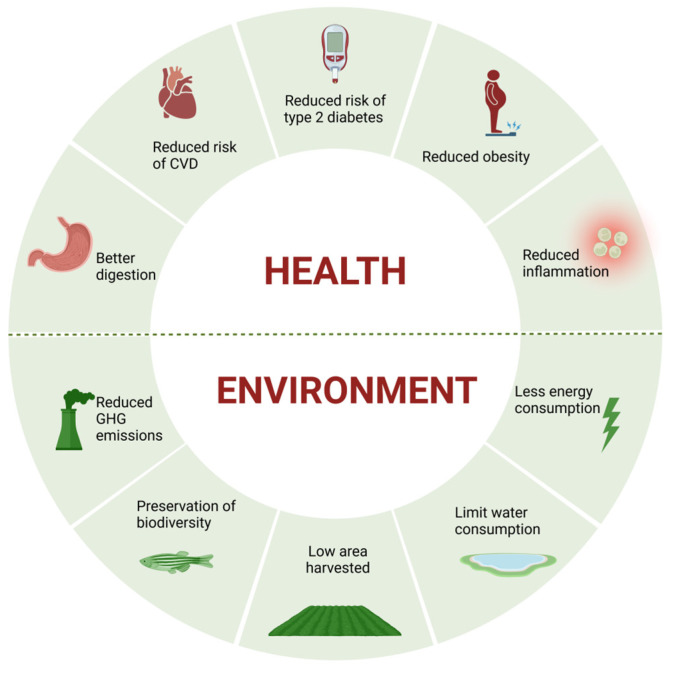
Plant-based diet benefits for health and environment (created with BioRender.com). CVD cardiovascular disease; GHG greenhouse gas.

Alongside the beneficial environmental outcomes, there are additional health advantages as well. Vegetarians have a lower risk of all-cause mortality, especially in relation to fatalities at younger ages and specifically for those linked to renal failure, infectious illnesses, diabetes, certain cardiac conditions, and ischemic heart disease [20]. Figure 1 illustrates the key health and environmental benefits of a vegetarian diet.

However, not all plant-based diets provide similar health benefits. Vegetarian diets lacking in certain nutrients (vitamin B12, iron, zinc, and calcium) and/or abundant in highly processed and refined foods elevate morbidity and death rates [21]. Regarding calcium levels, vegans have an inadequate calcium intake not just because they exclude dairy products but also owing to the bioavailability difficulties related to the calcium in plant-based meals. Nevertheless, the calcium incorporated into food items, such as certain kinds of tofu, exhibits comparable bioavailability to that of milk [22].

Strict vegetarian diets throughout pregnancy correlate with a heightened risk of small-for-gestational-age newborns and reduced birth weights. To reduce these concerns, precise dietary guidelines highlighting the sufficient consumption of essential nutrients, including vitamin B12, iron, omega-3 fatty acids, and iodine, should be established. Supplementation and customized dietary guidance, including fortified foods and plant-based sources, such as legumes, nuts, seeds, and algae-derived omega-3, are vital for maternal and newborn health [23].

Research indicates that meticulously structured vegetarian and vegan diets, supplemented adequately with vitamin B12, EPA, and DHA, are suitable during pregnancy and lactation, positively influencing the nutrients’ concentration in breast milk [24].

### 2.1. Health—Supporting Effects

Diet significantly influences general health and the management of chronic conditions, such as Alzheimer’s, diabetes, obesity, and cardiovascular illnesses. Moreover, dietary choices affect the inevitable aging process in both healthy persons and those with pre-existing health conditions. Research on animals has shown that caloric restriction can markedly delay the development of illnesses and potentially extend longevity. Furthermore, obesity has been associated with a faster aging process, as demonstrated by the prevalence of several key indicators of aging in obese persons. In addition, a well-established plant-based diet has a positive impact on atherogenic particles and lipoprotein(a), as well as inflammatory indicators [25].

Comprehending these linkages can provide insights into the interplay between food, aging, and chronic illnesses [26].

The key benefits of a plant-based diet include its beneficial impact on intestinal microbiota (due to higher fiber consumption), as well as its cardiovascular, neurological, and metabolic effects. Embracing this dietary regimen positively influences the preservation of bone health and the prevention of specific types of cancers.

#### 2.1.1. Gut Microbiota

Dietary fibers are a category of substances that provide various health advantages, due to their functional properties. They mostly consist of carbohydrates, particularly polysaccharides, including cellulose, hemicellulose, pectin, and resistant starch. In contrast to sugars and starches, the complex structure and chemical properties of dietary fiber make it resistant to digestion through the enzymes found within the small intestine. As a result, dietary fiber remains unchanged once it reaches the colon, serving as a substrate for gut microbiota [27,28]. Also, the intake of fiber-rich meals, such as barley, wheat bran, and brown rice, alongside fructo-oligosaccharides and similar prebiotics, is known to enhance butyrate-producing microorganisms [19].

Recent developments in laboratory methods have elucidated the roles of the human gut microbiota in relation to immunity and the gastrointestinal, neurological, and cardiovascular systems [29]. The bacterial fermentation of dietary fiber within the gastrointestinal tract enhances the synthesis of short-chain fatty acids, yielding advantageous effects on inflammation, lipid and glucose metabolism, and the integrity of the gut and blood–brain barriers [30].

A recent study identified specific differences in fecal bacteria diversity and composition between vegetarians and omnivores. A high-fiber vegetarian diet may enhance fecal bacteria diversity. An omnivorous diet high in fat and calories may diminish fecal bacteria diversity and is likely to contribute to being overweight or obese [31]. The short-term intake of animal-based products augmented the prevalence of bile-tolerant microbes while diminishing those that metabolize dietary plant polysaccharides, with a long-term impact on gut microbiota enterotypes [31,32]. Furthermore, diets based on plants support the establishment of more diversified and stable microbial organisms that are helpful to the health of humans [33].

However, there is still a need for furthering our comprehension of the impact of vegetarian and plant-based dietary habits on the gut microbiome, along with the metabolic changes that are associated with them, which are factors that contribute to disease processes.

#### 2.1.2. Cardiovascular

The primary cause of mortality and the reduction of the optimal life expectancy of humans is cardiovascular disease (CVD). It significantly contributes to health deterioration and elevated healthcare costs, imposing substantial pressure on global health systems. Consequently, it is essential to investigate the associated risk indicators promptly to mitigate the incidence of CVD [34].

Multiple research studies indicate that the plasma and urine concentrations of trimethylamine N-oxide (TMAO) serve as a valid biomarker for a CVD risk. Multiple studies investigating the association involving nutrition and TMAO concentrations in plasma or urine suggest that diets based on plants (Mediterranean, vegetarian, and vegan) successfully decrease TMAO levels, but animal-based diets show a detrimental effect [35].

A meta-analysis concluded that a vegetarian diet provides significant health advantages by reducing the probability of cardiovascular illness, along with ischemic cardiovascular disease; however, it has no impact on the stroke risk [36]. Compared to omnivorous diets, veganism appears to be favorably correlated to alterations in the risk indicators for CVD, including the total serum cholesterol, serum glucose, inflammatory processes, and blood pressure levels [37]. Research indicates that dietary patterns exclusively derived from plant sources are more efficacious in lowering blood pressure compared to other vegetarian diets that incorporate animal products, such as eggs and milk products, exemplified by the lacto-ovo-vegetarian diet. This finding has shown that the increased consumption of vegetables and fruits is associated with a lower blood pressure, in contrast to the finding in those who consume more dairy products [38].

These outcomes have significant public health implications as CVD remains the primary cause of mortality and morbidity worldwide, indicating that embracing plant-based dietary patterns, like vegetarianism, may be effective in lowering the burden of cardiovascular diseases [36].

#### 2.1.3. Neurologic

In the last ten years, vegetarianism has garnered considerable interest in the field of social neuroscience. Multiple studies have examined the correlation between vegetarianism and motivation, empathy, and cognitive attributes. The integration of MRI (magnetic resonance imaging) methods has facilitated the characterization of the psycho-cognitive profiles of vegetarians compared to those who consume meat. The current data indicate that vegetarians exhibit the distinct functional recruitment of networks associated with social mirroring, emotional contagion, and self-representation [39]. Compared to non-vegetarians, vegetarians were less likely to develop hemorrhagic, ischemic, and overall strokes [40].

Research indicates that vegetarianism does not correlate to depression when accounting for demographic factors and the level of education, body mass index, C-reactive protein levels, and smoking habits [41]. These diets, characterized by a high carbohydrate-to-protein ratio, could boost tryptophan transport into the brain, resulting in increased serotonin synthesis and reduced depression [33].

Exceeding that, researchers noted a decreased prevalence of dementia or Alzheimer’s disease in a substantial cohort from two religious groups that follow a lacto-ovo-vegetarian diet and adhere to certain lifestyle recommendations [42]. These findings are significant, considering the current discourse over the mental health dangers and advantages associated with vegetarian eating [41].

#### 2.1.4. Bone Health

Bone health is influenced by foods like soy and fruits and vegetables, as well as minerals like vitamin D, vitamin K, potassium, and magnesium. Fruits and vegetables that are rich in potassium and magnesium produce an alkaline ash that inhibits bone resorption. In premenopausal women, the higher bone mineral density of the lumbar spine and femoral neck is associated with potassium intake. Soy isoflavones have been shown to improve bone health in postmenopausal women. The soy isoflavone genistein substantially increased the bone mineral density of the lumbar spine and femoral neck compared to a placebo in a 24-month randomized clinical study involving osteopenic postmenopausal women [43].

A study revealed that in a healthy middle-aged population with a normal bone mineral density, an increase in plant food intake, whether alone or alongside a meat-inclusive diet, correlates with enhanced bone mineralization indicators. This correlation is likely due to the abundant micronutrients and phytochemicals included in plants, which have been demonstrated to significantly enhance bone health [44]. Phytoestrogens may induce both estrogenic and antiestrogenic events in the brain–pituitary–gonadal axis and peripheral reproductive organs. Legume seeds, a primary source of phytoestrogens, are essential elements of plant-based diets. These can improve bone density and decrease the risk of osteoporosis by modulating estrogen metabolism [45]. Alongside phytoestrogens, foods based on plants are rich in antioxidants, including carotenoids, which reduce the oxidative stress associated with aging and postpone bone loss in the elderly. According to a recent study, a vegetarian diet characterized by a high intake of potassium-rich nutrients was associated with a low dietary acid load, which is associated with lower bone resorption, thus promoting bone health [46].

A systematic review and meta-analysis of 18 observational studies involving 12,543 women indicated that the increased consumption of fruits and vegetables correlates with a reduced risk of postmenopausal osteoporosis, with reductions of 32% and 13%, respectively. A recent systematic review and meta-analysis assessed 13 publications from six cohort studies (involving 225,062 subjects) and four randomized controlled trials, indicating that a diet abundant in fruits and vegetables correlates with a reduced risk of bone fractures [47].

#### 2.1.5. Metabolic

Plant-based diets may be effective in preventing weight gain and managing body weight. For example, vegetarians exhibited a reduced body weight in comparison to non-vegetarians in cross-sectional research. Additional research indicated that a strong adherence to a plant-based diet is correlated with reduced body obesity [48]. Low-fat vegetarian and vegan diets were linked to substantial decreases in weight and plasma lipid levels, as well as decreased dietary advanced glycation end-products. In research that included changes in medication, a low-fat vegan diet showed superior effects on glycemia and plasma lipids compared to standard diabetic dietary guidelines [49,50]. An increase in postprandial incretin, along with insulin production, was seen after a serving of a plant-based tofu burger, suggesting the therapeutic value of plant-based meals for increasing beta-cell function in people with type 2 diabetes [51]. Veganism is associated with a decreased prevalence and incidence of type 2 diabetes, and in these patients, it lowers elevated glucose levels and enhances glucose homeostasis [52].

The lacto-ovo-vegetarian diet has been connected to a decreased risk of metabolic syndrome and insulin resistance and may have metabolic and cardiovascular protective effects in women [53]. A meta-analysis including 30 observational studies involving over 10,000 people indicates that diets rich in vegetables correspond to decreases in the total cholesterol, LDL cholesterol, and non-HDL cholesterol levels [30].

#### 2.1.6. Cancer

A vegetarian diet includes several nutritional components that protect against cancer. Fruits and vegetables are characterized as preventive against lung, oral, esophageal, and gastric cancers, with reduced efficacy against cancers at other sites, whereas the consistent consumption of legumes offers protection against gastric and prostate cancers. Moreover, dietary components such as fiber, vitamin C, carotenoids, flavonoids, and other phytochemicals demonstrate protective effects against several malignancies, while *Allium* vegetables provide shields from gastric cancer and garlic offers protection against colorectal cancer. Foods abundant in lycopene, like tomatoes, are recognized for their protective effects against prostate cancer [43].

Following an average monitoring period of 11.4 years, 54,961 new cancer cases were recorded, involving 5882 colorectal cancers, 7537 postmenopausal breast cancers, and 9501 prostate cancers. In comparison to normal meat consumers, those who were low meat-eaters, fish-eaters, or vegetarians had a reduced risk of all cancers. A modest meat consumption was linked to a reduced risk of colorectal cancer compared to normal meat consumption. The risk of breast cancer in postmenopausal vegetarian women was reduced; however, this risk diminished and became non-significant after controlling for body mass index [54].

Similar results were achieved by researchers who conducted research into the Adventist cohort, comprising over 70,000 participants, over an extension period of around 4 years; they revealed risk decreases for malignancies among lacto-ovo-vegetarians of −25% for gastrointestinal cancers and among vegans of −34% for female cancers and −16% for all cancers [30].

### 2.2. Environment—Supporting Effects

The food we consume significantly affects the environment [55]. Before increasing social awareness regarding the environmental impact of various dietary choices, the evaluation of many possibilities for achieving a sustainable and nutritious diet must incorporate relevant data regarding the nutritional value of diverse dietary practices [56].

Climate change, driven by manmade greenhouse gas emissions, is a significant current hazard to human health and the planet. Willett and colleagues assert that food is “the single strongest lever to optimize human health and environmental stability on Earth” [57]. Several global studies indicate that dietary modifications, including decreasing the intake of animal-derived foods and embracing vegetarian alternatives, can mitigate environmental impacts [58]. A diet consisting solely of plants, as a sustainable dietary approach, aligns with established goals for reducing emissions of greenhouse gases (GHG), the consumption of water, land use, nitrogen and phosphorus application, and chemical pollution, while also fostering biodiversity conservation [18].

The ecological footprint of an omnivorous diet is 2.57 and 2.8 times larger than that of vegetarian and vegan diets, respectively. This indicator includes all crops, grazing land, woodland, and fishing grounds necessary for food production, waste absorption, and infrastructure provision. Moreover, the water footprint of a diet high in meat (the total volume of freshwater utilized for production) exceeds that of vegetarian and vegan diets by more than three times [59]. Switching from the conventional Western dietary to a more environmentally responsible eating routine, which involves reducing or eliminating animal-derived products, may potentially reduce water use in food production by half and decrease land use and greenhouse gas emissions by up to 80% [59].

As regards packaged food, the environmental indicators of sustainable products are evaluated from both the sourcing and manufacturing perspectives of production. The primary indications for ingredient sourcing are to reduce GHG emissions, enhance soil quality, restrict synthetic pesticides, and foster biodiversity. Incorporating effects on downstream water quality is an essential indicator, considering the substantial advantages that sustainable sourcing may provide in this regard [60].

The ecological effects on terrestrial and aquatic ecosystems vary among the three dietary regimes. Water consumption is higher in lacto-ovo-vegetarian diets and omnivorous diets, attributable to the inclusion of animal-derived proteins. In summary, the increased consumption of protein from animals in a diet correlates with elevated water usage. A diet exclusively comprised of plant-based foods has the most potential for decreasing world water use. Furthermore, animal husbandry utilizes 70% of all agricultural areas and one-third of arable land. In conclusion, a completely plant-based diet exerts the minimal environmental effect [55].

## 3. Food Contaminants

Food pollutants, such as heavy metal ions, foodborne viruses, pesticides, mycotoxins, and antibiotics, have emerged as insidious threats to human life and health via dietary consumption and bioaccumulation. Food pollutants can induce acute or chronic poisoning and possess considerable carcinogenic, teratogenic, and mutagenic effects [61]. Persistent organic pollutants (POPs) are substances that resist degradation and disseminate readily in the environment [62]. More than 90% of human exposure to these pollutants occurs by ingesting contaminated food products, especially products derived from animals. Fish provide a primary source of exposure to these chemicals. To enhance public health protection, it is essential to comprehend the paths of POPs entering food, with the environment being a major conduit [63].

Moreover, certain food products, such as honey, may be contaminated with drug residues at varying levels, including chloramphenicol, sulfonamides, macrolides, nitroimidazoles, and nitrofurans, despite the fact that these compounds should not be present in food [64,65,66,67].

**Figure 2:**
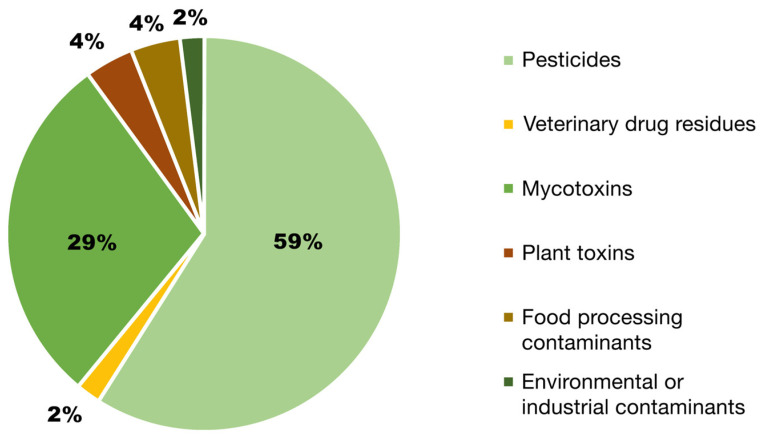
Percentage of food alerts documented from January 2020 to June 2023. Modified and adapted after Casado et al. [68,69].

According to research, one of the most common dietary contaminants is pesticide residues [62]. To support this statement, Figure 2 shows that pesticide residues represented the primary origin of food alerts in the EU in recent years, accounting for about 60% of total alerts, followed by mycotoxins, which constituted 29% of the total signals issued. Human exposure to mycotoxin-contaminated products mostly occurs via the intake of these products, potentially resulting in severe health issues, including immunosuppression and carcinogenesis [7].

Conversely, the number of food warnings in other pollutant categories were markedly reduced, and aquatic contaminants exhibited the lowest prevalence (0.4% in all food alerts). Every group of chemical pollutants is further detailed below, emphasizing the primary chemicals that trigger food warnings within these categories [68]. A significant proportion of recent food alerts about pesticide presence in food has been associated with organophosphorus pesticides, including chlorpyrifos and dimethoate. The food alerts highlight the presence of many pesticides, including acetamiprid, prochloraz, tricyclazole, carbendazim, and dimethoate, which are often found in food items at alarming concentrations [69].

Pesticides are categorized by their chemical structures into synthetic types (organophosphates, organochlorines, pyrethroids, neonicotinoids, and carbamates) and those derived from natural plant sources [62]. In recent decades, pesticides like DDT and dieldrin have been widely used in agriculture to increase crop output as well as to eliminate unwanted pests [63]. Owing to the hazardous characteristics of these compounds, several pesticides, including DDT and more recently glyphosate (organophosphorus), have been prohibited in various nations. Furthermore, in certain areas of Africa, DDT continues to be employed for the control of malaria larvae, while glyphosate is utilized for agricultural reasons throughout Latin America and the United States [70].

Following the harvest of raw agricultural commodities, the amount of pesticide residues in food is mostly determined by subsequent storage, handling, and processing. If proper agricultural and manufacturing practices are rigorously enforced, pesticide residues will be reduced to below the maximum residual level [71]. But irrational consumption sometimes results from farmers’ noncompliance with Good Agricultural Practices (GAPs) and inadequate pesticide management, including adherence to label-specified mixing concentrations and pre-harvest intervals. Farmers in various low- and middle-income countries do not comply with GAPs, owing to their circumstances [9].

### 3.1. Incidence in Food Products

The potential contamination with residues of pesticides by consuming fruits and vegetables is substantial. The Standard American Diet exposes individuals every day to a combination of several pesticides. These pesticide pollutants, despite their modest concentrations, are now acknowledged to contribute to nearly all chronic diseases [72]. Toddlers, particularly newborns, are more vulnerable to contamination owing to their lower body weight as well as higher food intake compared to adults, which is responsible for the increasing worry over food safety for this age group in recent decades. Infants and young children are considered especially susceptible populations, since fruits and vegetables constitute the first food offered during weaning and form the foundation of their solid diet for a minimum of two years [73]. Furthermore, during food processing, residual pesticides transform into other metabolic byproducts that are more hazardous than the original pesticides [74].

The Hazard Analysis and Critical Control Point (HACCP) system is regulated by two departments of the US government: the Food and Drug Administration (FDA) and the Department of Agriculture (USDA). The principal objective of HACCP is to reduce biological, chemical, and physical hazards to an acceptable level by detecting possible risks in food processing and instituting controls at critical points where hazards can be removed [75]. Comparable to the principles of human health, HACCP prioritizes prevention rather than treatment [76]. The proper management of food along the whole supply chain is required to guarantee food safety [77]. HACCP must effectively identify possible food safety issues proactively and establish control measures prior to the realization of these hazards [78]. While HACCP is not a fundamental component of GAP, it functions in conjunction with GAP, and in agricultural operations, GAP serves as the basis for HACCP [79].

Following national control programs, the European Commission has pointed out specific food items, fundamental to the diet, for monitoring pesticide residues since 2009. Residue level variations are monitored by mandatory coordinated multiannual control programs [23]. The European Food Safety Authority (EFSA) evaluates the outcomes of national and coordinated monitoring programs [80]. Recently, multiple studies were performed to quantify pesticide residues in food items to ensure compliance with regulatory standards. Table 1 delineates a number of studies carried out across various global areas, indicating that several tested samples exhibit pesticide levels above the legal maximum limits.

**Figure 3:**
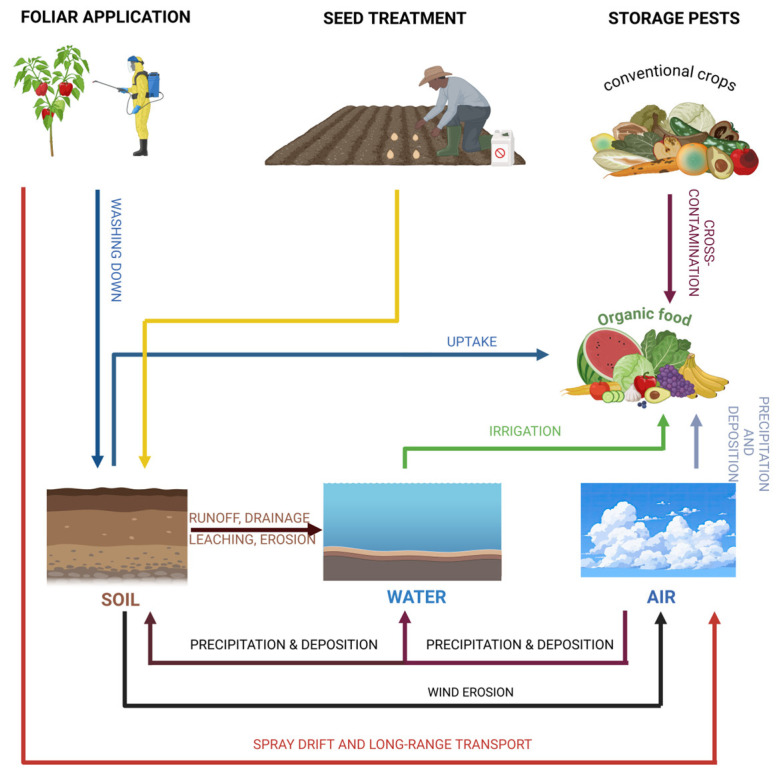
Pesticides distribution processes (created with BioRender.com). Modified and adapted after [93].

The European Commission defines organic production as a comprehensive system of agricultural management and food production that integrates optimal environmental practices, a significant level of biodiversity, the conservation of natural resources, and the implementation of rigorous animal welfare standards (European Parliament, 2018) [92]. The regulations governing organic agriculture involve several subjects, including agricultural cultivation, livestock management, food processing, soil conservation, and biodiversity protection. The organic industry seeks to reduce the contamination of organic products with such compounds, in accordance with consumer expectations (EU, 2018/848) [93]. Regarding the progress in organic agriculture, current studies have uncovered that several food products and soils within organic agricultural systems across various regions of the globe have been contaminated by pesticides [94,95]. Environmental migratory mechanisms transfer pesticide residues to different environmental compartments, including organic fields, where they persist for varying durations, as presented in Figure 3 [93].

**Figure 4:**
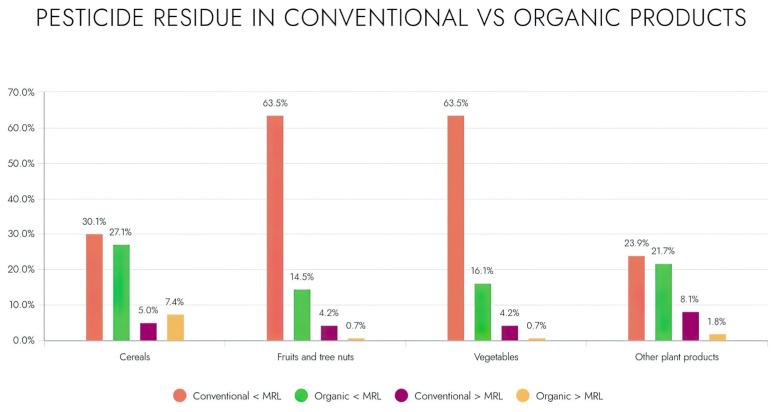
Pesticide residues below and above MRL in organic and conventional products.

In recent decades, organic farming has emerged as a prominent subject of discussion in global agriculture. This is due to the customer demand for organic products rather than conventional ones. Consumers prefer organic products despite their greater cost and substantially inferior production, owing to the absence of the harmful chemicals utilized in conventional agriculture [96]. Organic farming comprised 8.5% of all EU agricultural land in 2019, a 66% increase since 2009 [94]. Organic food is usually considered to be safer, healthier, and more environmentally sustainable, with a superior nutritional content and reduced levels of harmful compounds [94,95,97]. Figure 4 illustrates the prevalence of pesticide residues in organic compared to conventional goods, according to the study provided by the EFSA in 2022 [98].

In contrast to conventional agriculture, the yields of organically cultivated crops are frequently much lower, hence impacting profitability and adoption rates. To enable the shift to organic farming, measures that reimburse farmers for opportunity costs are frequently essential [99]. Many challenges, such as time, biotic stressors, availability, and product marketing, must be collectively addressed, necessitating the participation of various disciplines to establish a viable organic farming [100]. Furthermore, organic farming approaches may decrease weed density without significantly affecting diversity and evenness. The absence of adequate skills and information regarding appropriate weed control technologies among farmers has contributed to the persistent presence of weeds in organic farms. To implement a successful weed control strategy, the farmer requires a comprehensive understanding of the practices to apply [101].

Despite its reduced yields, the environmental performance of organic farming is typically seen as equivalent to that of conventional farming when assessed on a per-kilogram basis. The comparative economic performance of organic farming vs. conventional agriculture remains contentious, taking into account productivity, reduced input costs, emerging market prospects, and diminished risk exposure owing to more consistent yields over time [102].

Authorities establish the maximum allowed levels for pesticide residues in conventional agricultural products; however, no specific maximum limits have been set for organic products. Both organic and conventional foods must follow the maximum residue limits set out in Regulation (EC) No. 396/2005. Article 5 of Regulation (EC) No. 889/2008 on the organic production of agricultural goods highlights limitations on the utilization of plant protection agents. In 2022, the rates of MRL exceedance were lower in organic food compared to conventionally produced food across all the analyzed product categories. In 2022, 6.717 samples designated as organic food (excluding baby food) were analyzed, representing 6.1% of the total samples. Overall, none of the 5305 samples that were labeled as organic showed any detectable residues (79.0% of the analyzed samples compared to 82.8% in 2021); 1.252 samples contained quantified residues at or below the MRL level (18.6% versus 15.4% in 2021); and 160 samples were identified with residue levels exceeding the MRL (2.4% versus 1.8% in 2021), of which, 1.4% (92 samples) were deemed noncompliant. The pesticides most commonly identified over the limit of quantification (LOQ) but below the maximum residue limit (MRL) were copper compounds (RD) at 78.3%, bromide ion (RD) at 14.0%, and chlorates (RD) at 8.6%. The herbicide exhibiting the greatest maximum residue level exceedance rate was copper compounds (RD) at 11.9% [103].

### 3.2. Harmful Effects on Environment

Toxic substances can be detected in air, water, soil, plants, food, and animal feed. These residues permeate vegetation and animal products, accumulating in both humans and animals via the food chain. They jeopardize our lives and compromise our overall well-being while also obliterating advantageous creatures within the environment [104]. Alaoui et al. revealed that the majority of residual pesticides discovered in the soil, agricultural products, water, and sediment, including illegal substances nonetheless used for diverse applications, have been identified as potentially harmful to ecosystems and human health [105].

The main components of the agroecosystem are soil, water, plants, and crops. These components keep the ecosystem lively and viable by dynamic interactions among themselves. Global concerns are heightened due to the decline in soil biodiversity and food safety, exacerbated by pesticide contamination. All pesticides are active agents that provide risks and dangers [104]. Pesticides have water solubility and thermal stability, which can extend their hazardous impact on the environment [62].

The use of pesticides in the environment has a wide range of consequences on both target and non-target species. Many pesticides exhibit a broad spectrum of biological activity that surpass the specific purposes indicated by the producer. This is attributable to their effects and the unintended or non-target actions of certain chemicals that may alter pathogen activity, hence affecting disease severity induction [70].

Pesticides used in farming have the potential to travel through the atmosphere and land in other parts of the environment, such as soil and water. When pesticides are sprayed on the ground, they may be carried away by surface runoff and end up in nearby sources of water, or they may seep into the soil and eventually into groundwater [106]. Wind and water facilitate the transfer of polluted soil, disrupting ecosystem functioning, while other exposure pathways may affect non-target organism health. Soils are significantly contaminated and appear to subsequently infect the crops cultivated within them [105]. Research carried out in Europe on 76 pesticides revealed the presence of residues in surface soil throughout the continent. A total of 83% of the soil samples had residues, and 58% of those samples had two or more residues. Glyphosate and its metabolites were consistently found at the highest concentrations. A wide variety of pesticides has been identified in rivers, lakes, and surface water throughout Europe [106].

Pesticides travel via water, polluting water resources. Pesticide contamination of both surface water and groundwater pose a severe and pressing hazard for freshwater and coastal ecosystems across the world [107]. According to the United States Geological Survey, more than 90% of water and fish samples collected from US streams contained one or more pesticides [104].

Additionally, pesticides may negatively impact beneficial soil microorganisms by inducing metabolic alterations in these species [11].

Pesticides are reducing the populations of animals, including marine mammals, alligators, fish, and piscivorous birds. It is believed that the deaths of thousands of Arctic seals are linked to the buildup of persistent chlorinated hydrocarbons, including DDT, polychlorinated biphenyls (PCBs), and dioxins, in the food chain. These substances accumulate in adipose tissue and compromise the immune systems of animals. It is believed that the deaths of striped dolphins in the Mediterranean, beluga whales across the Saint Lawrence River, and sea lions from the Pacific Ocean are attributable to the buildup of harmful contaminants [4]. Historically, substances like DDT and several organochlorine compounds were considered to have minimal acute toxicity to animals. Nonetheless, their application resulted in occurrences such as eggshell thinning and the bioaccumulation of organochlorines in the fat tissue of long-lived species, prompting the emergence of less-persistent chemicals, such as organophosphates and carbamate insecticides [11].

Concerns over the impact on aquatic life and the health effects on humans associated with chlorpyrifos and diazinon have initiated a transition from these organophosphate insecticides to pyrethroid insecticides, characterized by reduced water solubility and less toxicity to mammals. This transformation has diminished the impact of organophosphates on both water quality and overall human toxic effects; however, extensive research reveals increased detections and the aquatic toxic effects of pyrethroids in sediments downstream from agricultural areas, notably the marine waters that receive them [108].

Despite it being an illegal act in European Union countries, the deliberate poisoning of animals, both domesticated and wild, remains widespread for the purpose of managing hazardous species in several nations. Moreover, domesticated animals are frequently poisoned by pesticides. Reports indicate that around 52.5% of birds are poisoned by pesticides, which are also a primary source of mortality in wild mammals [104].

Plant biodiversity promotes the populations of bees and other pollinator insects. However, herbicides diminish plant communities and negatively impact birds, mammals, fish, insects, amphibians, and reptiles. The United States Fish and Wildlife Service (USFWS) estimates that one-fifth of honeybee colonies in Europe have been destroyed by pesticides. The bees are endangered when (1) they are enveloped in spray drifts; (2) agrochemical residues are detected in pollen, honey, water, hives, larvae, and feed; and (3) when the combs treated with acaricidal chemicals expose the bees to these substances [104].

Addressing the ecological consequences of chemical pollution from agricultural practices necessitates protecting wildlife from existing chemical contaminants, removing these pollutants if feasible, and bolstering efforts to avert additional contributions to terrestrial and aquatic ecosystems [109].

### 3.3. Harmful Effects on Human Health

The main ways that pesticides come into contact with individuals are through the food chain, the air, water, soil, flora, and wildlife [110]. Upon absorption into the human body, they are transported in the circulation throughout the gastrointestinal tract, urinary tract, integumentary system, and respiratory tract. The cutaneous, oral, ocular, and respiratory routes are the primary paths for pesticide penetration into the human body [104]. Pesticide exposure by ingestion was determined to be more detrimental than dermal contact, rendering the consumption of raw fruits and vegetables from unidentified sources potentially hazardous [111].

**Figure 5:**
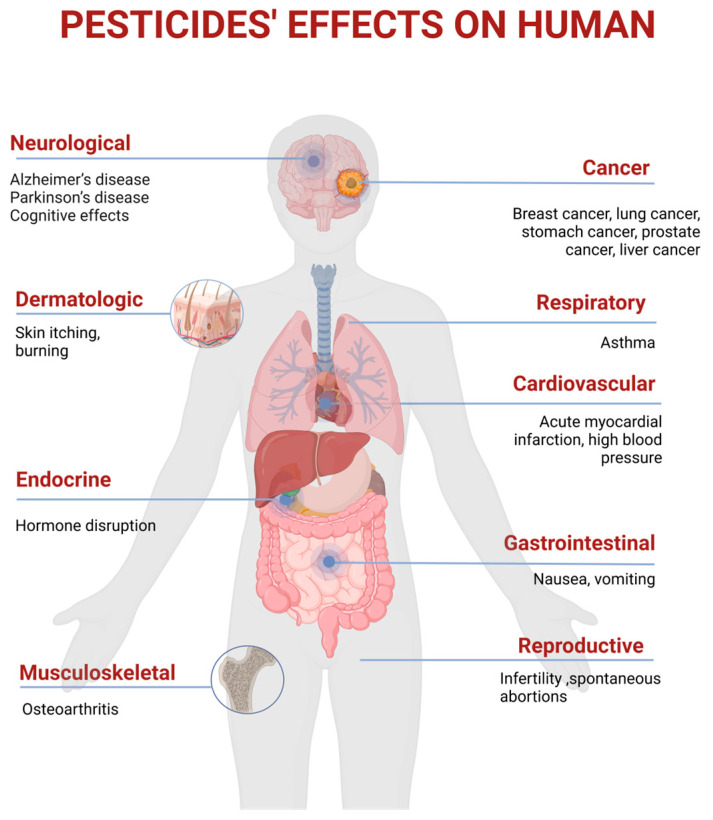
Pesticide residue toxicity in human body (created with BioRender.com).

An estimated 385 million episodes of accidental acute pesticide poisoning occur each year, resulting in around 11,000 fatalities. Approximately 44% of farmers worldwide—roughly 860 million people—suffer from pesticide poisoning each year. Approximately 20,000 people in developing nations perish from pesticide-contaminated food each year [11]. Exposure to these pollutants may result in several health issues, including endocrine disruption, cardiovascular illnesses (acute myocardial infarction, high blood pressure), malignancies, different types of cancer (breast cancer, lung cancer, stomach cancer, prostate cancer, liver cancer, urinary bladder cancer, leukemia, non-Hodgkin lymphoma, brain tumor), diabetes, birth abnormalities, impaired immunological and reproductive systems, Alzheimer’s disease, and Parkinson’s disease, as shown in Figure 5 [63,112,113,114]. Studies indicate the cumulative impact of concomitant exposure to numerous pesticides may influence human health considerably more than isolated exposures [112].

The Environmental Protection Agency (EPA) prohibited pesticides, such as DDT and ethylene dibromide, due to their alarming carcinogenic and mutagenic effects [11]. The latest evidence about the toxicity of these substances is presented in Table 2.

## 4. Pesticide Removal from Fruits and Vegetables

The presence of pesticides in food can be significantly decreased through industrialized and household methods of preparation, such as washing, blanching, peeling off, and thermal treatment. Typically, home processing removes residues in a synergistic way [124].

Processing foods is an extensive procedure which modifies both the chemical and physical characteristics of food [125]. Food processing techniques may mitigate health concerns linked to pesticide exposure by decreasing pesticide residues in food items [74]. It is essential to assess the degree of exposure at the moment of ingestion and post-cooking [124]. In recent years, several innovative thermal as well as non-thermal techniques have been designed to reduce chemical residues in fresh produce [126]. The efficacy of pesticide residue removal is mainly determined by both the chemical and physical characteristics of the pesticides, particularly their solubility [125].

### 4.1. Household

Since most vegetables are consumed raw, home processing methods, such as washing, peeling, and boiling, are necessary to reduce the residual pesticides on them [90]. In practical applications, easily accessible household solutions, particularly sodium carbonate, might be used with tap water to reduce pesticide residues on fresh food [127]. A recent study evaluated the efficacy of various harmless cleaning agents, including tap water and various quantities of Na_2_CO_3_ (sodium carbonate), NaCl (sodium chloride), C_2_H_4_O_2_ (acetic acid), ACV (apple cider vinegar), and GVS (grape vinegar solutions), for residues of buprofezin, thiophanate-methyl, imazalil, and abamectin in oranges. The efficacy hierarchy of the treatments was as follows: alkaline solutions (10% Na_2_CO_3_) > acidic solutions (8% C_2_H_4_O_2_) > vinegar solutions ≈ neutral solutions (10% NaCl) > tap water [127].

Concerns have arisen over the potential for commercial detergent washing to facilitate the deeper penetration of pesticide and surfactant residues, leading to secondary contamination. Reports indicate that detergents can cause endocrine disruption in the human body during prolonged accumulation [128]. Therefore, washing with non-toxic chemical solutions, such as acetic acid, sodium carbonate, and sodium chloride, might be considered safe treatments [127].

Considering cooking methods, studies suggest that various cooking techniques provide different impacts on pesticides. For instance, the residual concentration of acetamiprid increased in green chilis following boiling and stir-frying [124]. Of the two domestic processing techniques for teff flour, baking is more efficient in reducing residues than fermentation. The domestic processing procedures of doughing and baking were responsible for the decrease of residues, as shown by the PF (processing factor) value [129]. In addition, pre-soaking rice in extra water prior to its preparation is the most effective approach for reducing pesticide residues [130].

Li et al. conducted a thorough investigation of processing parameters, along with their correlation with physicochemical attributes, of 13 pesticides in field-collected pepper samples throughout the production of Chinese chopped pepper and chili powder. The processing parameters were washing (W), air drying (AD), chopping and salting (CS), and fermenting (F), which decreased the remaining amounts of 13 pesticides by 24.8–62.8% for W, 0.9–26.4% for AD, 25.1–50.3% for CS, and 16.3–90.0% for F. The processes of drying them in the sun and crushing amplified the residues of 11 pesticides, with a factor of 1.27 to 5.19 [131].

### 4.2. Industrial

Washing, the initial phase in both domestic and commercial food preparation, contributes to reducing pesticide residues on the surfaces of fruits. Industrial washing applications, particularly ozone treatment, chlorine treatment, and electrolyzed water, have been described as efficient procedures for eliminating the residues of pesticides [127]. The use of non-toxic chemical solutions, such as acetic acid, sodium carbonate, and sodium chloride, has been explored for their efficacy in eliminating pesticide residues from the surfaces of distinct fruits and vegetables [127].

Ozone, being a strong oxidant, possesses a significant capacity to eliminate these residues from food. Because of its antimicrobial properties, ozone not only minimizes the effects of pesticides but also improves the quality and shelf life of food. This environmentally friendly, non-thermal technique has garnered extensive support among researchers and experts. Nonetheless, the processing protocols must be standardized for various types of agricultural products. Consequently, it is essential to monitor any issues encountered in the application of ozone technology and to identify solutions for its sustainable utilization [132].

In 2002, electrolyzed water was introduced as a sustainable food sterilizing and disinfection technique in Japan. Following that, other studies examined the disinfection efficacy of electrolyzed water on the pathogens present in raw or freshly cut vegetables. Washing with electrolyzed water was demonstrated to be better for pesticide removal from fresh-cut vegetables in contrast to conventional washing solutions. In a study, Liu et al. demonstrated that alkaline electrolyzed water (AlEW) is the most suitable washing solution for eliminating pesticides from colored peppers, whereas acidic electrolyzed water (AcEW) proved to be more successful for both cabbage and broccoli florets [126].

The use of ultrasonic waves at frequencies ranging from 20 to 100 kHz has garnered the attention of researchers as an effective non-thermal physical cleaning technique. Ultrasound is a recently discovered potential technique that effectively eliminates remaining pesticides, dyes, and drugs, amongst others, with only minor substrate degradation and without generating secondary by-products or contaminants [133]. In contrast to laborious and ineffective conventional cleaning procedures that frequently need substantial quantities of chemical disinfectants, the ultrasound-assisted washing of fruits and vegetables is rapid, effective, and efficient. It can eliminate pesticide residues and other impurities while preserving the color, nutrition, and texture of fresh fruit. It is a cleaning method that can be simultaneously time-efficient and energy-effective. This method has been evaluated on several fruits and vegetables, such as grapes, cabbage, carrots, tomatoes, and cucumbers. Ultrasound-assisted cleaning is recognized as an eco-friendly and efficient technology for pesticide elimination that is distinguished by its superior capacity to remove pollutants, relative to traditional techniques. It is an efficient approach for conserving time and energy throughout the cleaning process [134,135].

In comparison to conventional water washing, an ultrasonic washer proved to have superior efficacy in pesticide elimination, achieving clearance rates of 14.7% to 59.8% for rapeseed and 72.1% to 100% for grapes. Nonetheless, notable discrepancies existed in the removal rates of five pesticides on rapeseed and grapes [136].

The triple-frequency sequential mode was selected as the optimum method for pesticide elimination, reaching clearance rates of 92.31%, 89.36%, and 95.25% for abamectin b1, alphamethrin, and emamectin benzoate, respectively, after 8 min of ultrasonication. The impact of ultrasound on physiological and nutritional characteristics indicates that the quality of triple-frequency sequential mode-treated lettuce is equivalent to that of water-washed food, as evidenced by the total chlorophyll and total carotenoid levels. Observations from scanning electron microscopy indicate that the triple-frequency sequential mode causes minimal surface damage to lettuce [137].

## 5. Sustainable Approaches for a Healthier Plant-Based Diet

A food system that is sustainable supports global nutrition and food safety while maintaining the socioeconomic and environmental foundations vital for future generations [138]. Whenever feasible, alternative and more sustainable methods should be investigated to achieve a balance between crop protection and environmental preservation [11]. In scenarios of high pesticide application, even in the absence of health hazards to consumers from pesticide residues, farm operators should be informed and urged to seek guidance from a plant protection expert. This expert would assess the prevailing growing conditions during the season and provide guidance on the efficient and cost-effective use of pesticides [80].

Understanding the significance of policy and regulation reveals that a comprehensive approach—integrating scientific progress, economic incentives, and legal structures—is essential for altering food production and consumption patterns towards a healthier and more sustainable future. Although policy integration in environmental and rural sectors started about thirty years ago, emerging concerns related to food and nutrition security and shifting priorities necessitate more integration [139]. Sustainable diets and food systems play an essential role in attaining the global sustainable development objectives. Consequently, governments are being asked to implement substantial modifications to food policy that realign food production and consumption in support of these [18].

The responsible utilization and investigation of alternative approaches is essential for alleviating the adverse impacts of conventional pesticides [11]. In order to reduce the inappropriate use of synthetic pesticides and minimize food contamination from residues, alternative and sustainable pest management approaches are being recommended, including Integrated Crop Management (ICM), the utilization of biopesticides, and the implementation of the IPM (Integrated Pest Management) strategies that integrate biological, physical, and chemical approaches, thereby diminishing reliance on chemical pesticides.

Unfortunately, the transition to sustainable agriculture encounters considerable challenges in underdeveloped nations, as economic and institutional limitations restrict access to alternative methods. One study indicates that, in certain areas, the obstacles to revolutionary changes are significant in terms of economic, financial, and institutional factors [140]. Most sustainable agricultural initiatives in underdeveloped countries are financed by donors and lack mechanisms for sustainability once project funding ceases. The majority of smallholder farmers consistently encounter a multitude of problems and limits associated with various concerns, mostly stemming from limited resources (land, water, capital, personnel) and restricted access (knowledge, information, inputs, technology, opportunities). In these conditions, the significance of economic incentives in promoting sustainable agricultural management cannot be overstated [141]. Moreover, access to education is an essential aspect in reducing population growth, which, therefore, influences future food demand and agricultural land utilization [142].

### 5.1. Integrated Pest Management (IPM)

IPM is an environmentally sustainable approach to pest control that utilizes a combination of techniques, including chemical, cultural, and biological treatments [143]. The techniques used include biological control and crop rotation [11].

Implementing an IPM system will reduce the adverse effects of pesticides on the ecosystem and human health while maintaining agricultural output and minimizing the danger of crop losses [106].

**Figure 6:**
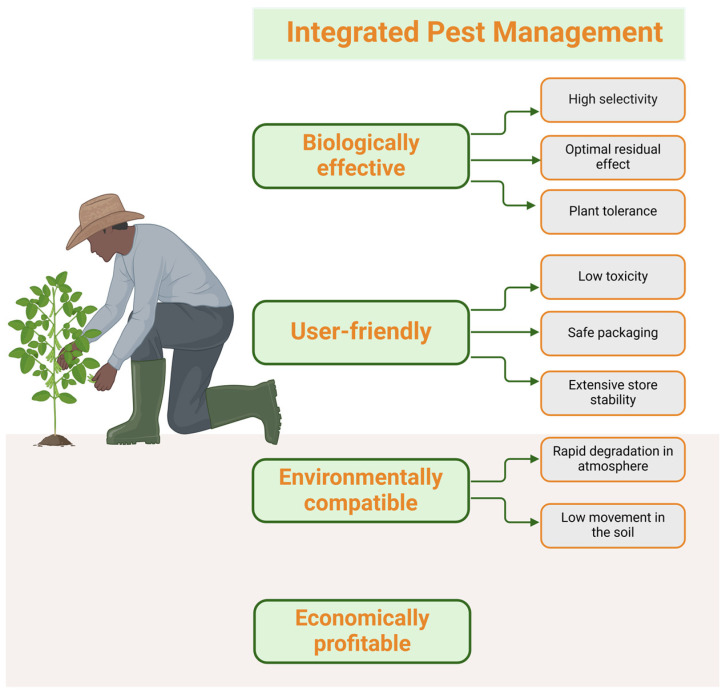
Criteria for selecting pesticides in IPM (created with BioRender.com).

An investigation performed in Nepal revealed that the overall acceptance of Integrated Pest Management (IPM) in growing vegetables is 37%, while the majority of farmers are lagging behind in the application of IPM technology, underscoring the need for ongoing dissemination initiatives. Enhancing access to educational materials, data, and alternative approaches to pest management empowers farmers to make informed decisions and effectively use IPM strategies [144]. The criteria for selecting pesticides in IPM are presented in Figure 6.

### 5.2. Integrated Crop Management (ICM)

Agricultural experts initiated the formulation of alternative crop management approaches that might reduce the adverse environmental and health consequences of pesticide use in crop protection. ICM provides directives for farmers to use practices that ensure the production of safe agricultural products while maintaining environmental sustainability. Moreover, ICM includes recommendations for the successful implementation of Good Agricultural Practices (GAPs), worker sanitation and protection, product security, the thorough traceability of measures, and the focused preservation of environment initiatives. Integrated Crop Management enhances the implementation of complementary pest management strategies, such as crop resistance to fungus and insects, biological pest management, and safeguarding protocols, while reducing the detrimental effects of these chemicals on other components in the agroecosystem. ICM allows pesticide application exclusively through an IPM program that employs precise criteria for pesticide selection, adheres to detailed application instructions for crops, and utilizes residue analysis as a regulatory measure [106].

### 5.3. Biopesticides

Biopesticides are pesticides found in nature or that are derived from living organisms or their metabolites. In short, a biopesticide is a product that mimics the activity of a natural substance while still possessing pesticidal qualities. The US EPA divides biopesticides into three broad categories depending on the sorts of bioactive chemicals or agents employed for pest control: (1) biochemical pesticides, (2) microbial pesticides, and (3) plant-incorporated protectants [145]. Biopesticides provide significant potential as they not only control pests but also reduce the adverse impacts of synthetic chemical pesticides [146].

Biopesticides, as green agrochemicals, significantly impact sustainable agriculture by following the principles of green chemistry and the threefold concept of sustainable development. Furthermore, the inter-relationship between sustainable development and green chemistry in biopesticide-driven commercial agriculture guarantees environmental preservation, enhanced production both quantitatively and qualitatively, safe and efficient technology, and cautious resource utilization [147]. In the near future, biopesticides could substitute synthetic pesticides with no substantial impact on productivity and yield, if their potential is fully utilized [148].

#### 5.3.1. Biochemical Pesticides

Biochemical biopesticides are chemicals, or specific synthetic analogs, derived from natural sources that include active components that manage pests through methods that are both safe and non-toxic to the intended pest, the ecosystem, and people [147]. Examples include insect sex pheromones (which interfere with reproduction and species expansion), various fragrance compounds (which lure insect pests into traps), as well as certain vegetable oils [149]. For instance, many essential oils derived from plants in the *Cupressaceae* family have been evaluated for their insecticidal and acaricidal properties [150]. Research shows the essential oils of *Chamaecyparis lawsoniana* essential oil and *Thuja plicata* essential oil function as antibacterial agents against a number of harmful bacteria and fungi. These essential oils have significant potential as sources of antibacterial and antigenotoxic substances, as well as prospective biocides against pest insects and arthropods [151].

#### 5.3.2. Microbial Pesticides

The utilization of biological pesticides presents an optimal alternative that is safe, cost-efficient, easily adoptable, and effective against many insect pests and diseases. Insects, similarly to other creatures, can contract a diverse array of illnesses caused by numerous microorganisms, including bacteria, fungus, viruses, protozoa, and nematodes [152].

Entomopathogenic bacteria (EPB) are unicellular organisms that may infect and eliminate arthropods, such as insect pests and mites. Many bacterial species of EPB possess the capability to induce infection and inflict harm on insects. The application of EPB has been implemented inside the IPM program [152]. *Bacillus thuringiensis* (Bt) dominates 90% of the microbial biopesticide industry; yet, *Steinernema*, *Beauveria bassiana*, *Nosema*, and *Chlorella* have additionally shown considerable influence [147]. A significant decrease in Acetylcholinesterase enzyme activity in larval tissues was observed with *Bacillus thuringiensis* treatments (58.8%) compared to untreated control groups [153].

#### 5.3.3. Plant-Incorporated Protectants (PIPs)

PIPs are genetically modified plants which offer resistance to pest infestations. The developments in recombinant DNA and plant transformation technologies have transformed commercial agriculture for pest management by successfully incorporating insect management agents into derived from plants protectants. The insecticidal compounds utilized in PIP technology include Bt Cry proteins, toxic complex proteins from *Xenorhabdus* and *Photorhabdus*, α-amylase inhibitors, protease from Baculovirus, double-stranded ribonucleic acid (dsRNA), and Mir1-CP from maize [147].

In planta delivery offers significant benefits over conventional spray or soil application methods, such as a lesser environmental impact, targeted efficacy against specific pest insects, and streamlined, continuous protection throughout the season. The possibility of resistance to insects, which has limited the efficacy of traditional exogenous pesticides, remains an obstacle for PIPs [154].

### 5.4. Microbial Assisted Remediation

Microbial-assisted remediation, or bioremediation, is an eco-friendly procedure that employs microorganisms to decompose or neutralize pollutants in contaminated areas, such as soil, water, and air.

Microorganisms, such as bacteria, fungi, and algae, are used in microbial-assisted remediation to decompose or alter pollutants, including pesticides, in polluted settings. Pesticides are metabolized through these bacteria into less harmful or non-toxic substances through the process of detoxification, biological degradation, and bioaccumulation. This technology can entirely decompose pesticides under optimal conditions, proving to be both cost-effective and ecologically friendly. It is particularly beneficial in areas near the coast, where chemical contamination by urban centers and agricultural wastewater is prevalent [143].

The capacity for biodegradation relies on enzymes such as oxidoreductases, monooxygenases, dioxygenases, carboxylesterases, phosphotriesterases, haloalkane dehalogenases, haloalkane dehydrochlorinases, diisopropylfluorophosphatase, paraoxonase, and organophosphate acid anhydrolase [155]. Enzyme-mediated bioremediation can be classified into phase I and phase II; the former enhances the solubility of pesticide compounds via oxidation–reduction and hydrolysis reactions, whereas the latter converts toxic pollutants into less toxic or non-toxic products through conjugation reactions [156]. Vaithyanathan et al. studied the use of biosolids for laccase production by the inoculation *of Pleurotus dryinus*. The crude enzyme extract containing laccase derived from sterilized biosolids bioaugmented with *P. dryinus* demonstrates the extensive specificity of *P. dryinus* for pesticides and their efficient removal, highlighting its potential as a bioremediation agent without the necessity for supplementary mediators [157]. Recent advancements in enzyme-mediated bioremediation encompass immobilization, encapsulation, and protein engineering, which enhance stability, recyclability, handling, storage, and reaction control [156]. Regulating and enhancing these processes can augment the efficacy of food cleanup, rendering microbial remediation a viable option for the contemporary food industry.

## 6. Conclusions and Perspectives

This research explored new evidence suggesting the benefits of a vegetarian diet. Current food consumption practices in developed countries are detrimental in terms of human health and the ecosystem. As a meat-centric diet exacerbates environmental challenges and provides health concerns to humans, promoting a plant-based diet is vital for attaining vegetable production and availability.

In addition, concerns regarding pesticide residues in various food products were addressed, determining the risk associated with the ingestion of these chemicals. Identifying pesticide residues in plant-derived foods is essential for protecting human well-being and the planet. Despite the overall sustainability and health benefits linked to a diet based on plants, the contamination of vegetables and fruits with pesticides represents a significant concern. Additional research is necessary to ensure the continuous monitoring of pesticide residues in food products, given the possible dangers to human health and the environment, which could vary based on the specific uses and limitations of pesticides. Further study is required to consistently evaluate the effects of pesticide residues on ecosystems and to develop novel techniques for their detection and mitigation, taking into account emerging agricultural practices and changes in the environment that may affect pesticide levels.

The research comprehensively evaluated the detrimental ecological consequences of pesticides and ultimately recommended environmentally friendly practices for their reduction or removal, as well as environmentally suitable alternatives to pesticide use. This study can facilitate the development of straightforward, user-friendly guidelines to assist in attaining national and international objectives for public health and environmental sustainability. Moreover, enhancing the public knowledge of pesticide residue presence and the associated dangers may encourage a transition to more sustainable food production practices that emphasize human health and environmental integrity.

To conclude, stakeholders must undertake definitive efforts to advance the transition to sustainable food systems. Farmers may implement agroecological measures, like IPM and crop diversity, to decrease dependence on chemical inputs. Additionally, consumers play a vital role in supporting sustainably produced products and minimizing food waste, whereas governments need to establish incentives, assistance, and regulatory frameworks that foster ecologically sustainable agriculture approaches.

## Figures and Tables

**Table 1:** Pesticide residues in fruit and vegetables.

Reference	No. of Samples	Location	Commodity	Outcomes
Loughlin et al. 2018 [81]	135	Argentina	Fruits and vegetables	65% positive for at least one pesticide <MRL: 29% >MRL: 36%
Zhang et al. 2023 [82]	573	China	Citrus fruits	81% positive for two or more PR >MRL: 9.4%
Yun et al. 2024 [83]	223	South Korea	Soybean, persimmon, mandarin, peach, potato, pepper, radish, Korean cabbage, apple, brown rice, onion, and tomato	All samples contain levels < MRL
El-Sheikh et al. 2023 [84]	124	Egypt	Tomato, strawberry, and their products	20 pesticides detected. 15 pesticides detected in tomato sauce. 8 pesticides exceeded MRLs.
Balkan et al. 2023 [85]	48	Turkey	Tropical fruits	PR detected in 41.7% of samples All samples contain levels < MRL
Muhammad Arif et al. 2021 [86]	360	Pakistan	Raw milk	>MRL: γ-HCH and β-endosulfan
Balkan et al. 2022 [87]	74	Turkey	Leafy vegetables	13 different PR detected LOQ-MRL: 57.6% >MRL: 6.75%
Ambrus et al. 2023 [80]	9924	Hungary	Fruits, vegetables, cereals, baby food	<LOQ: 45.94% LOQ—MRL: 53.01% >MRL: 1.03%
Gomez et al. 2023 [88]	226	Turkey	Mandarins	<LOQ: 8.4% >MRL: 22.1% 40 different residues detected
Elgueta et al.2021 [89]	57	Chile	Fresh tomatoes	>MRL: 9% Residue free: 39%
Ramadan et al. 2020 [90]	211	Saudi Arabia	Tomato, cucumber, cabbage, eggplant, chili pepper, onion, potato, carrot, lettuce, and cauliflower	Residue free: 31.3% >LOD: 68.7% <MRL: 47.9% >MRL: 20.9%
Ouakhssase et al. 2024 [91]	30	Morocco	Filet green beans	8/30 green bean samples are positive. Azoxystrobin—most frequently detected pesticide. Residue of fipronil—unauthorized substance—detected in one sample at 0.027 mg/kg. All samples contain levels < MRL.

MRL—maximum residue limit; PR—pesticide residue; LOQ—limit of quantification; LOD—limit of detection.

**Table 2:** Toxicological effects of pesticides.

Reference	Toxic Effect	Age Group	Outcomes
Albadrani et al. 2024 [115]	Spontaneous abortion risk (SAB)	Pregnant women aged ≥ 16	Induction of inflammation, oxidative stress, disruption of endocrine function. 41% increase in the risk of SAB among pregnant women exposed to pesticides.
Araki et al. 2018 [116]	Alterations in reproductive hormones within umbilical cord blood	Infants	Maternal exposure to OCPs ↓ steroid hormone levels among infant boys. OCP exposure → sex differences. Low levels of exposure to OCPs in utero → alter steroid hormones at birth.
Miao et al. 2021 [117]	Breast cancer	Women aged > 30	Urinary HNE-MA + 8-isoPGF2ɑ.
Miani et al. 2021 [118]	Autism spectrum disorder	Children	Gestational exposure to certain organophosphate agents. Pyrethroid exposure during pregnancy or early life.
Fuhrimann et al. 2021 [119]	Impaired visual memory	Adults (farmers)	Link between glyphosate exposure and visual memory in farmers.
Zhu et al. 2024 [120]	Osteoarthritis	Adults aged > 20	OPP exposure in patients with ASCVD. Urine dialkyl phosphate metabolites ↑↑.
Islam et al. 2023 [121]	Respiratory and allergic effects	Children 5 years old	Pyrethroid and mancozeb exposure. LRTI. Asthma. Symptoms of eczema.
Caba et al. 2022 [122]	Poisoning	Children: 1–5 years old (*n* = 31); 6–11 years old (*n* = 6); 12–17 years old (*n* = 11).	48 cases reported. Toxic encephalopathy (*n* = 21). Coma (*n* = 8). Depressive conditions (*n* = 5). Gastrointestinal conditions (*n* = 13). Respiratory failure (*n* = 6).
Sarno et al. 2024 [123]	Local and systemic symptoms	Children 6–14 years old	Italian children living close to cultivations sprayed with pesticides. Often—eye/skin/lower airway/systemic symptoms. Daily—upper airways symptoms, systemic symptoms.

HNE-MA—4-hydroxy-2-nonenal-mercapturic acid; PG—Prostaglandin; OPP—organophosphorus pesticide; ASCVD—Atherosclerotic Cardiovascular Disease; LRTI—Lower respiratory tract infections; ↓ means reduced; ↑↑ means high levels.

## Data Availability

The data are contained within this article.
